# Pediatric dentists who accept new Medicaid-enrolled children report higher willingness to advocate for community water Fluoridation

**DOI:** 10.1186/s12903-019-0812-7

**Published:** 2019-06-14

**Authors:** Vinodh Bhoopathi, Anna Vishnevetsky, Jennifer Mirman

**Affiliations:** 1 Department of Pediatric Dentistry and Community Oral Health Sciences, Temple University Maurice H. Kornberg School of Dentistry, 3223 N Broad Street, Philadelphia, Pennsylvania 19140 USA; 2 3700 Market Street, Suite 101, Philadelphia, Pennsylvania 19104 USA; 3 Temple University Maurice H. Kornberg School of Dentistry, 3223 N Broad Street, Philadelphia, Pennsylvania 19140 USA

**Keywords:** Fluoridation, Advocacy, Oral health advocacy, Medicaid, Social responsibility, Rural dentists, Altruism, Pediatric dentists

## Abstract

**Background:**

Dentists, who advocate for Community Water Fluoridation (CWF), can help decrease the dental caries disparity gap between low and high socioeconomic groups. Advocating for CWF, a cause that promotes oral health at the population level is an altruistic behavior. Dentists who accept and provide services to Medicaid-insured children, who are from low socioeconomic backgrounds, are also considered altruistic. We tested the association between accepting new Medicaid-insured children every month, and willingness to advocate for CWF programs in pediatric dentists (PDs).

**Methods:**

In 2016, a 22-item pilot tested online survey was sent to 5394 PD members of the American Academy of Pediatric Dentistry. Descriptive analysis and a multiple adjusted logistic regression model was conducted.

**Results:**

Dentists who accept new Medicaid-insured children every month (OR: 1.62; 95% CI: 1.06–2.47; *p* = 0.02) were more willing to advocate for CWF compared to their counterparts. Those practicing primarily in rural (OR = 4.67; 95% CI: 1.82–11.9; *p* = 0.001), and urban areas (OR = 2.27; 95%CI: 1.05–4.89; *p* = 0.04), and those willing to promote fluoridated water consumption to parents in the clinic (OR = 3.40; 95% CI: 1.87–6.21; p = < 0.0001) were significantly more likely to be willing to advocate for CWF. PDs trained in public health advocacy during pediatric residency alone (OR = 2.37; 95% CI: 1.24–4.51; *p* = 0.009), or during both pre-doctoral dental education and pediatric residency (OR = 3.51; 95% CI: 1.87–5.6; p = < 0.0001) were more willing to advocate for CWF compared to their counterparts.

**Conclusions:**

PDs who accepted new Medicaid-insured children every month were more willing to advocate for CWF programs compared to those who did not.

## Background

Community water fluoridation (CWF) was introduced in 1945 and has since been noted as one of the ten great public health achievements of the twentieth century [1]. CWF is “the controlled addition of a fluoride compound to a community water supply to achieve a concentration optimal for dental caries prevention” [2]. CWF, is a cost effective, safe, socially equitable, population based approach that reduces dental caries among all population groups [3]. CWF can decrease the disparity gap in dental caries levels between high and low socioeconomic groups [3]. A study in Northern England showed that socioeconomic status (SES) and water fluoridation influenced dental caries experience [4]. They found a greater disparity in dental caries between high SES and low SES groups in non-fluoridated communities compared to fluoridated communities [4].In another study, there was noticeable disparity in age-standardized dental caries mean values between SES groups living in a non-fluoridated area compared to the groups living in a fluoridated area [5]. The disparity between the groups was higher in non-fluoridated area, with low SES groups having a higher mean dental caries compared to high SES groups [5]. In a 2018 report released by Public Health England on water fluoridation, it was concluded that children from all areas benefited from drinking fluoridated water, but children from relatively deprived areas benefited the most [6]. These data show that CWF has a greater impact in reducing the dental caries experience in people from low socioeconomic backgrounds, and minimizes the disparities in dental caries between higher and lower SES groups.

The Department of Health and Human Services’ Oral Health objective 13 (OH-13) of the Healthy People 2020 goals sets a 2020 goal of increasing the proportion of the US population served by community water systems (CWS) with optimally fluoridated water to 79.6% [7]. Water fluoridation statistics from the Centers for Disease Control and Prevention (CDC) for the year 2014 reveal that 90% of the US population was served by CWS, however less than 75% of those served with CWS actually received fluoridated water [8]. While millions of people in the US lack access to fluoridated water, antifluoridationists tirelessly work to hinder CWF initiatives, and to defluoridate existing fluoridated communities. As dental health experts and credible sources for oral health information, dentists have a huge responsibility to step forward and clear misconceptions about CWF [9].

Dentists can educate their patients about the benefits of consuming fluoridated water in their clinical practice. They also can proactively advocate for CWF at the community and/or state level. Advocacy is defined as “to speak up, to plead, or to champion for a cause while applying professional expertise and leadership to support efforts on individual (patient or family), community, and legislative/policy levels, which result in the improved quality of life for individuals, families, or communities” [10]. Especially in low SES communities, dentists can promote consumption of fluoridated water and advocate for CWF programs. Consequentially dentists can be instrumental in reducing the disparities in dental caries between SES groups. In this regard, advocating for a cause that promotes oral health at the population level is an altruistic behavior, and thus dentists who advocate for CWF initiatives can be considered altruistic.

In a 1974 survey study conducted by the American Dental Association Research Institute of 4000 dentists, 85% of the responding dentists credited CWF as having a greater value in dental caries prevention, compared to other efforts [11]. In an another study, more than 90% of the responding dentists from Multnomah County, Portland, Oregon (1981) stated that CWF was desirable or highly desirable [12]. No recent studies have been conducted to understand the practicing dentists’ perceptions about CWF, let alone their willingness to promote fluoridated water consumption, or to advocate for CWF programs.

Advocacy is to speak out on behalf of a program or a population, and is involved in active promotion of a cause or principle [13]. We first aimed to understand the characteristics of pediatric dentists who were willing to advocate for CWF programs compared to those who were not willing. In the context of this study, advocacy would mean to support or speak for CWF to city councils, children’s organizations, and other public health organizations in their communities or states. We also tested the association between accepting new Medicaid-insured children every month in the clinical practice, and willingness to advocate for CWF programs among pediatric dentists. We were interested to explore this association because, the literature shows that dentists who accept Medicaid-insured patients in their practice have significantly more altruistic attitudes in general compared to those who do not [14]. Providing services to Medicaid children can be considered altruistic because these children are usually from low SES backgrounds and the reimbursement for dental services through Medicaid is substantially lower than through private dental insurance. Because advocating for CWF can be considered an altruistic behavior, we assume that dentists who accept new Medicaid-insured children every month are altruistic, and would also be more willing to advocate for CWF programs compared to those who do not. We also assessed the reasons behind the reluctance to promote fluoridated water consumption within clinical practice and/or reluctance in advocating for CWF programs at community or state levels.

## Methods

### Sample

Our target population were practicing pediatric dentists in the U.S. who were also active members of the American Academy of Pediatric Dentistry (AAPD). The study protocol was reviewed and approved by the Institution Review Board (Protocol Number: 23283).

### Survey instrument

A 22-item, pilot tested survey instrument was used to conduct this cross sectional study (A copy of the survey is provided in the appendix). The survey comprised of yes/no type questions, close ended and also questions to elicit open responses. Using cognitive interviewing techniques we pilot tested the survey with 5 pediatric dentists (PDs). To understand the dentists’ thought as they reviewed the survey, we adopted concurrent think aloud method with probes [15]. Piloting this survey using these methodologies we believe, enhanced the content and face validity of the survey [15].

PDs were asked several questions to determine their perceptions and willingness to promote the consumption of fluoridated water within their clinical practice and to advocate for CWF programs. Prior to asking questions about fluoridated water, a brief statement on CWF was included. The statement read: “Community water fluoridation (CWF) is the controlled adjustment of fluoride in a public water supply to optimal concentration in order to prevent dental caries among members of the community”.

First, we inquired whether PDs were likely to promote the consumption of fluoridated water when talking to patients in their clinical practice (Yes/No). Those who were unlikely to promote fluoridated water consumption were further asked about the reasons for their unwillingness. A checkbox list of possible reasons was provided in the survey, along with an open-ended option for respondents to state their own reasons. Another prompt statement said: “Some dentists have publicly expressed their support, and speak out for (advocate) community water fluoridation to city councils, children’s organizations, and other public health organizations in their communities or states”. This statement was included to inform the participants about the context in which CWF advocacy could occur. After this statement, PDs were asked whether they were willing to advocate for CWF programs (Yes/No). If they were not willing to advocate for CWF, we asked the reasons why they were unwilling. We then provided 5 different checkbox options (reasons) and an open-ended option to determine why respondents were unwilling to advocate for CWF. We determined whether PDs received any formal training in public health advocacy during their dental education by asking if they received any such training in their pre-doctoral dental education, or in their pediatric dental residency program, or during both pre-doctoral dental education and pediatric dental residency programs.

### Data collection

An online version of the survey instrument was created in Survey Monkey® (www.surveymonkey.com), an online web-based survey management tool. After checking the online survey for typological and operational errors, the survey was sent to 5394 PDs, along with 3 additional reminders to improve the participation rate. The AAPD provided a list of pediatric dentist members’ email addresses who were residing in the US at the time of the study. The online survey was open from mid-February 2016 until mid-May 2016. Of the 5394 pediatric dentists who were emailed, 385 opted out of the study. In addition, due to invalid email addresses the survey was not delivered to 139 PDs. The total number of PDs who responded to the survey was 830 (approximate response rate: 16%).

Standard guidelines recommended by Dilman et al. were used to improve the response rates [16]. Some of the strategies used were: 1) varying messages across reminders, 2) repeated contacts, and 3) determining if the online survey was compatible on different devices and softwares. The content of the email message was slightly changed without changing the meaning of the message during each reminders. We did this to vary the stimulus across each email contact. For repeated contact, we included: a) an introductory email informing the pediatric dentists of the upcoming survey, b) an email with a message about the purpose of the study with a personalized online link to access the survey, 3) reminder emails sent to both partial and non-respondents over a period of 2 months; and 4) three reminder emails. We used plain text message instead of HTML messages in the email to reduce the likelihood of the email being flagged as spam. We tested the compatibility of the online survey on iphones, androids, desktops, and different software and hard ware configurations.

### Statistical analysis

Descriptive analyses were performed to understand the study sample’s characteristics. In addition, we conducted a logistic regression model determining the characteristics of pediatric dentists who were willing to advocate for CWF versus not willing, after adjusting for confounders. Variables that were included in the adjusted multivariate logistic regression model include: age (years), gender (Male/Female), race (Whites/ Non-Whites), accepting new Medicaid-insured children monthly in clinical practice (Yes/ No), primary practice location (rural / suburban / urban [but not inner city]/ inner city) [17], willingness to promote consumption of fluoridated water in clinical practice (Yes/ No), and prior advocacy training during dental education (pre-doctoral education only / pediatric residency program only/ both pre-doctoral education and pediatric residency program/ no training).

## Results

The mean age of the participants was 43.7 ± 10.6 years. A majority of the responding pediatric dentists were females (57%) and reported belonging to a white racial background (78%) (Table 1). Approximately 65% of participants reported that they were currently accepting new Medicaid-insured children monthly. When asked about their primary practice location, most (58%) stated their primary practice was located in suburban areas, 22% in urban (not inner city) areas, 12% in rural areas, and 8% in inner city areas.

**Table 1:** Characteristics of the responding pediatric dentists

Variable	Frequency (%)^a^	Not responding per question (N)
Gender		6
Female	470 (57%)	
Male	354 (43%)	
Race		7
White	646 (78%)	
Black or African American	35 (4.2%)	
American Indian or Alaskan Native	2 (0.2%)	
Asian	101 (12.2%)	
Native Hawaiian or Pacific Islander	9 (1.1%)	
Other	36 (4.3%)	
Accepting new Medicaid-insured patients		27
Yes	523 (65%)	
No	280 (35%)	
Primary practice location		23
Inner city	65 (8%)	
Urban (not inner city)	176 (22%)	
Suburban	469 (58%)	
Rural	97 (12%)	

^*^ -All numbers may not add to a 100% because of missing responses

**Fig. 1:**
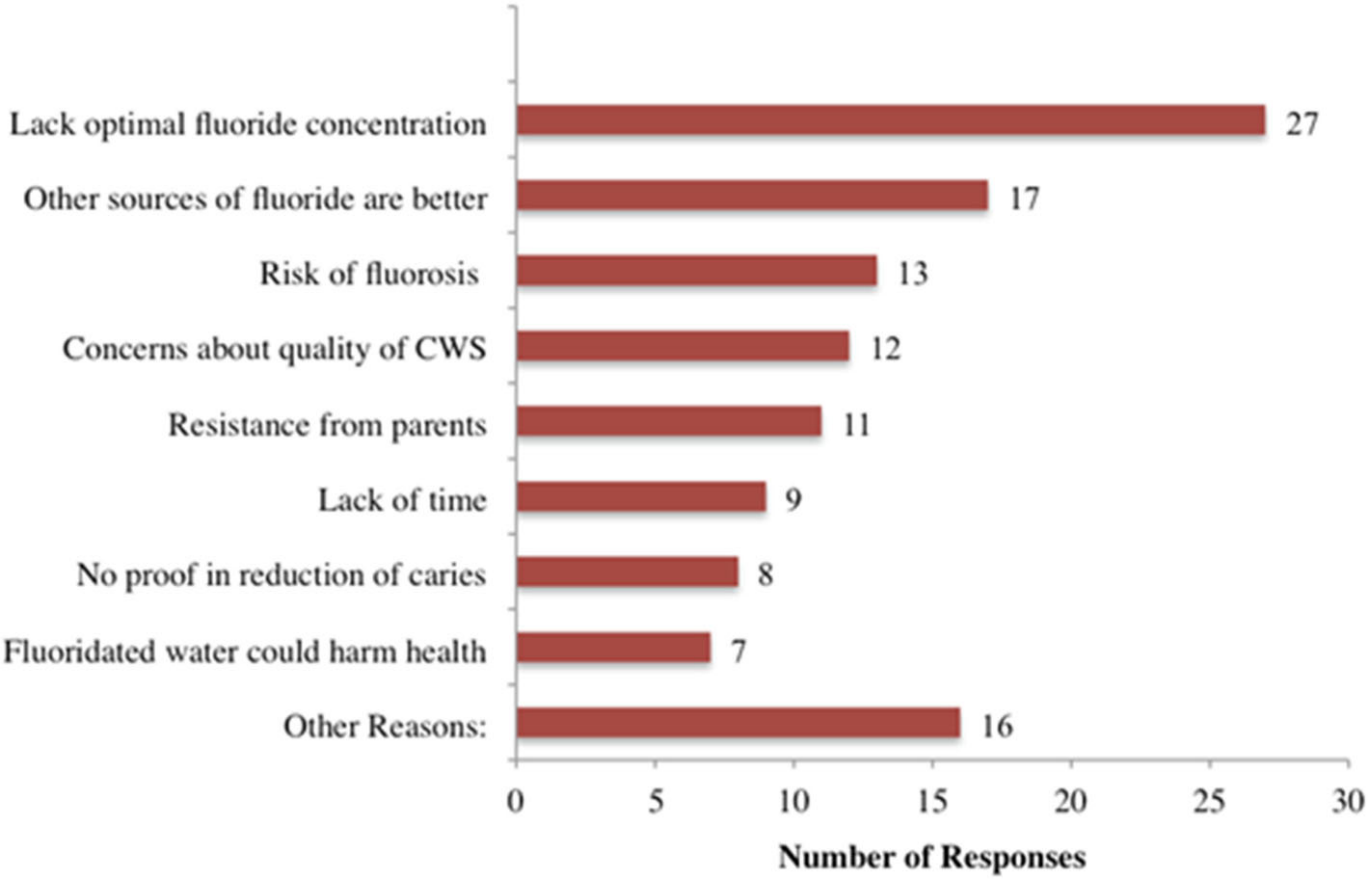
Reasons for being unlikely to promote fluoridated water consumption in clinical practice

As high as 90% of the respondents reported that they were likely to promote consumption of fluoridated water to children and parents in their clinical practice (Table 2). Of the 10% who reported that they were not likely to promote consumption of fluoridated water within their clinical practice, the most common reasons cited were: a) their community’s water system not being optimally fluoridated, b) the opinion that other sources of fluoride are better, c) the risk of fluorosis, d) concerns about the quality of community water systems, and e) parents’ resistance to adopt new behaviors (Fig. 1).

**Table 2:** Pediatric dentists’ responses to questions related to promoting and advocating for CWF and prior advocacy training during dental education

Variable	Frequency (%)^a^	Not responding per question N
Likely to promote consumption of fluoridated water in clinical practice		65
Yes	687 (90%)	
No	78 (10%)	
Willing to advocate for CWF at community or state levels		70
At both, community and state	481 (63%)	
At community, but not state	97 (13%)	
At state, but not community	10 (1%)	
Not willing to advocate	172 (23%)	
Prior Advocacy Training		73
During Predoctoral dental education only	48 (6%)	
During pediatric dental residency only	124 (16%)	
During predoctoral dental education and pediatric residency	164 (22%)	
No training at all	421 (56%)	

^*^- All numbers may not add to a 100% because of missing responses

**Fig. 2:**
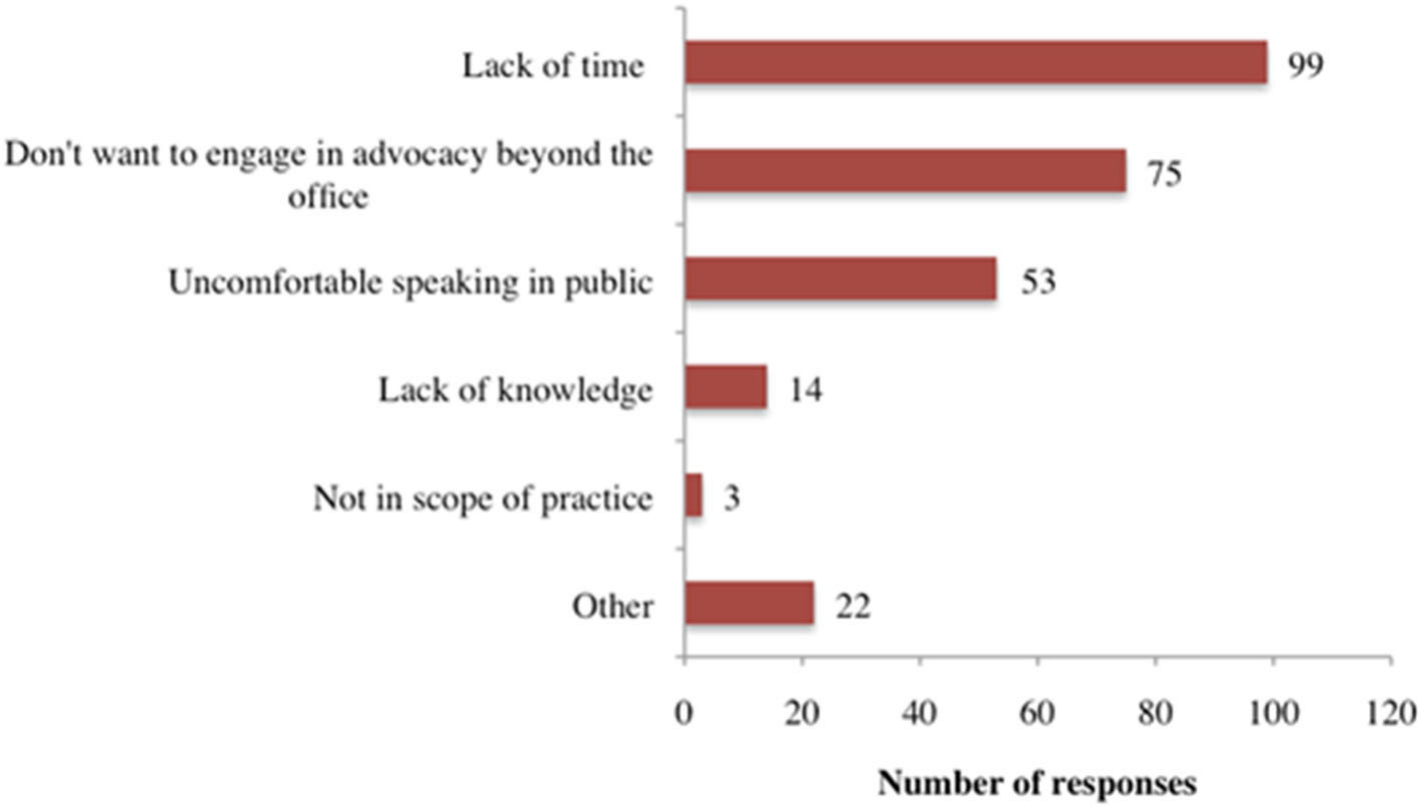
Reasons for not willing to advocate for community water fluoridation

Approximately 3 out of 4 respondents (77%) reported their willingness to advocate for CWF programs at community and/or state levels (Table 2). Those unwilling to advocate most commonly cited lack of time as the barrier to being advocates for CWF initiative. Other common reasons included not wanting to advocate beyond the dental office, and having public speaking anxiety (Fig. 2). Approximately 6% of respondents reported receiving advocacy training during their pre-doctoral education, 16% during their postdoctoral training, 22% during both pre-doctoral education and postdoctoral training, and more than half (56%) did not receive any training in advocacy during either pre-doctoral education or postdoctoral training (Table 2).

Logistic regression analysis (Table 3) showed no differences in willingness to advocate for CWF programs by age, gender, or race. Dentists who reported accepting new patients insured by Medicaid in their practice (*p* = 0.02), who were likely to promote consumption of fluoridated water in their practice (*p* < 0.0001), and those practicing primarily in rural (*p* = 0.001), or urban (not inner city) (*p* = 0.04) areas were significantly more likely to be willing to advocate for CWF. Additionally, those who were trained in public health advocacy during pediatric residency (*p* = 0.009), or during both pre-doctoral education and pediatric residency (p = < 0.0001) were significantly more likely to be willing to advocate for CWF compared to those who had not received training in public health advocacy.

**Table 3:** Multivariable adjusted logistic regression analyses of characteristics associated with willingness to advocate for CWF among pediatric dentists

Variable	Odds Ratio (95% CI)	*p*-value
Age (years)	1.01 (0.98–1.02)	0.79
Gender (Female Vs Male)		
Female	1.05 (0.69–1.61)	0.82
Male	REF	
Race (Non-Whites Vs Whites)		
Non-Whites	1.08 (0.65–1.79)	0.76
Whites	REF	
Accepting new Medicaid-insured patients every month (Yes Vs No)		
Yes	1.62 (1.06–2.47)	**0.02** ^*^
No	REF	
Primary practice location		
Rural	4.67 (1.82–11.9)	**0.001** ^*^
Sub-urban	1.52 (0.76–3.03)	0.23
Urban-not inner city	2.27 (1.05–4.89)	**0.04** ^*^
Inner city	REF	
Promote consumption of fluoridated water in clinical practice		
Yes	3.40 (1.87–6.21)	**< 0.0001** ^*^
No	REF	
Advocacy training		
Trained during predoctoral program	0.73 (0.35–1.54)	0.76
Trained during pediatric residency	2.37 (1.24–4.51)	**0.009** ^*^
Trained during predoctoral and pediatric residency program	3.51 (1.87–5.6)	**< 0.0001** ^*^
No training	REF	

^*^- Statistically significant

## Discussion

Many national and worldwide medical, dental, and public health organizations support CWF initiatives. Although CWF has had significant impact [1], dental caries remain prevalent in the U.S [18]. In 2015–2016, the total caries experience in children 2 to 19 years was approximately 46% [18]. The prevalence of total dental caries decreased as family income levels increased, from 56.3% for youths from families living below the federal poverty level to 34.8% for youths from families with income levels greater than 300% of the federal poverty level [18]. While substantial evidence exists supporting the effectiveness of CWF, water fluoridation initiatives are constantly threatened. For example, in the last few years, residents of communities have petitioned against CWF, and many counties or cities have stopped fluoridation throughout the US [19–21] perhaps because the public, city officials and lawmakers receive inaccurate information about the consequences of CWF from antifluoridationists’ social media sites [22, 23], and during city council debates. In these instances, dental professionals (especially dentists) can advocate for water fluoridation programs, and educate the public and governmental officials about the benefits of CWF programs.

In this study we surveyed the pediatric dentist members of AAPD who were actively practicing the profession in the US. It was encouraging to determine that most respondents (90%) were likely to promote consumption of fluoridated water in their clinical practice. This indicates that pediatric dentists, at least in our study, believe in the effectiveness and the importance of fluoridated water. However, when asked if they were willing to advocate for CWF, only 77% had a positive response. Almost 20% of the respondents who were unwilling to advocate cited that they were uncomfortable speaking in public. The high proportion of dentists stating willingness to advocate for CWF demonstrates a need for public health advocacy, and CWF issue related training programs to help dentists become better advocates. We recommend developing and disseminating a comprehensive oral health advocacy toolkit, which could provide a set of practical tools to educate interested dental professionals, including dental and dental hygiene students, about the different strategies, and modes to effectively advocate for important oral health and overall health issues.

Logistic regression modeling revealed some interesting and compelling findings. First, pediatric dentists who currently accepted new Medicaid-insured children every month were significantly more willing to advocate for CWF compared to those who did not accept new Medicaid-insured children. In a previous study, Iowa dentists who accepted Medicaid-insured patients demonstrated more altruistic attitudes, compared to those who did not accept Medicaid-enrolled patients [14]. Our study supports these results, if one considers willingness to advocate for a public health issue, like CWF, to be an altruistic attitude. Altruistic attitudes could be enhanced by educational and professional experiences such as service learning and/or community based clinical experiences in dental school [24].

Pediatric dentists who reported their primary practice as being located in a rural area were significantly more likely to be willing to advocate for CWF compared to dentists practicing in inner city areas. Though there is abundant literature on the differences between health care providers practicing in urban and rural areas, very little is known about their perceptions about advocacy or altruistic attitudes based on practice location. In a study of rural-urban differences of practicing physicians, rural primary care physicians worked longer hours, completed more patient visits, and accepted more Medicaid-insured patients compared to their urban counterparts [25]. Ours is the first study to identify the association between dentists’ practice location and their willingness to advocate for CWF programs.

In a previous 2011 study of AAPD members, 90% of the responding members thought that advocacy was an integral role of a pediatric dentist, but only 22% had received any form of advocacy training [26]. This study found that as high as 44% of the respondents had received some form of advocacy training, which is very encouraging. Pediatric dentists who received advocacy training during their pediatric residency program were more willing to advocate for CWF compared to those who did not (OR = 2.4, 95% CI: 1.2–4.5, *p* < 0.009). Those who received advocacy training during both their pre-doctoral dental education, and their pediatric residency training were the most willing to advocate for CWF (OR = 3.5, 95% CI: 1.9–5.6, *p* < 0.0001) compared to those who did not receiving any training. This indicates that repetitive exposure to advocacy training promotes further willingness to advocate. Data indicates that training at the pediatric dental residency level is sufficient to promote willingness to advocate for CWF, however, the data also suggests that it is desirable for advocacy training to be integrated into the predoctoral dental curriculum as well.

We acknowledge our study’s limitations. This study is a convenience sample and the response rate was very low, which limits its external validity and generalizability of findings. It would have been ideal to survey a random sample of dental clinicians across the US, however this was not realistic with our limited funds. The low response rate could have been improved had we approached the dentists by postal mail, yet this too was unfeasible due to our limited funds. Study participation was voluntary, and participants may have self-selected to be a part of the study, which may have led to self-selection bias. Therefore the participants in this study do not represent the entire pediatric dentist members of AAPD. A small sample size may be a concern, however the posthoc sample analysis showed that the final sample of 830 was sufficient to run a regression model with 7 predictor variables. Unfortunately, due to the anonymity of the survey we were unable to track who responded and who did not to determine the differences between the two groups.

CWF is a population-based preventive method, which can prevent the initiation and progression of dental caries. Pediatric dentists are respected health practitioners whose opinions about oral health are important to their patients and their communities. If pediatric dentists can promote fluoridated water consumption within their clinical practice, and advocate for CWF at the state and community levels, many people, especially from those in underserved and low SES communities will reap the benefits of CWF.

## Conclusion

A majority of the respondents were likely to promote fluoridated water consumption to their patients in clinical practice. In addition, every 3 in 4 responding PDs reported willingness to advocate for CWF programs at community and/or state levels. Dentists accepting at least 1 new Medicaid-enrolled child monthly were more willing to advocate for CWF programs compared to those who did not.

## Data Availability

Data cannot be shared at this time because more publications are planned using the same data. The survey used in this study is made available.

## Abbreviations

AAPD: American Academy of Pediatric Dentistry
CWF: Community Water Fluoridation
SES: Socioeconomic Status

## References

[1] Centers for Disease Control and Prevention (1999). Achievements in public health, 1900–1999: fluoridation of drinking water to prevent dental caries. MMWR.

[2] Department of Health and Human Services (US), Office of the Surgeon General (2000). Oral health in America: a report of the surgeon General.

[3] Burt B (2002). Fluoridation and social equity. J Public Health Dent.

[4] Provart SJ, Carmichael CL (1995). The relation- ship between caries, fluoridation and material deprivation in five-year-old children in county Durham. Community Dent Health.

[5] Kumar JV, Swango PA, Lininger LL, Leske GS, Green EL, Haley VB (1998). Changes in dental fluorosis and dental caries in Newburgh and Kingston, New York. Am J Public Health.

[6] Water Fluoridation: Health monitoring report for England 2018, Public Health England. Available at https://assets.publishing.service.gov.uk/government/uploads/system/uploads/attachment_data/file/692754/Water_Fluoridation_Health_monitoring_report_for_England_2018_final.pdf. Accessed 04.19.2019.

[7] Office of Disease Prevention and Health Promotion. Oral health: healthy people 2020. Available at: https://www.healthypeople.gov/2020/topics-objectives/topic/oral-health/objectives. Accessed 18 Apr 2019.

[8] Centers for Disease Control and Prevention. Community Water Fluoridation: 2014 Water Fluoridation Statistics. Available at: http://www.cdc.gov/fluoridation/statistics/2014stats.htm. Accessed 18 Apr 2019.

[9] Melbye ML, Armfield JM (2013). The dentist's role in promoting community water fluoridation: a call to action for dentists and educators. J Am Dent Assoc.

[10] Wright CJ, Katcher ML, Blatt SD, Keller DM, Mundt MP, Botash AS, Gjerde CL (2005). Toward the development of advocacy training curricula for pediatric residents: a national Delphi study. Ambul Pediatr.

[11] Dentists Credit Fluoride for Tooth Decay Reduction. Public Health Rep 1975; 90, p. 281. Available at http://www.jstor.org/stable/4595244. Accessed 04.19.2019.

[12] Isman R (1984). Knowledge and attitudes of dentists about fluoridation. JADA.

[13] KU Work Group for Community Health and Development. Chapter 3, Section 1: Developing a Plan for Assessing Local Needs and Resources: Community Toolbox Kit. Lawrence: University of Kansas; 2017. Available at http://ctb.ku.edu/en/table-of-contents/advocacy/advocacy-principles/overview/main. Accessed 8 June 2019.

[14] McKernan SC, Reynolds JC, Momany ET, Kuthy RA, Kateeb ET, Adrianse NB, Damiano PC (2015). The relationship between altruistic attitudes and dentists’ Medicaid participation. J Am Dent Assoc.

[15] DeMaio TL, Rothgeb J, Hess J (1998). Improving survey quality through pretesting.

[16] Dillman DA, Smyth JD, Christian LM (2009). Mail and internet surveys: the tailored design method.

[17] American Academy of Pediatrics (2012). Periodic Survey of Fellows No. 82.

[18] Fleming E, Afful J (2018). Prevalence of total and untreated dental caries among youth: United States, 2015–2016. NCHS data brief, no 307.

[19] David DeCamp. Pinellas County commission votes to stop putting fluoride in water supply. Available at: https://www.infowars.com/pinellas-county-commission-votes-to-stop-putting-fluoride-in-watersupply/. Accessed 8 June 2019.

[20] Hernando, Florida: Hernando commissioners say no to fluoride.. 2017-09-14. Available at: http://www.assureasmile.com/miami-dentist-blog/hernando-florida-commissioners-say-no-fluoride/. Accessed 8 June 2019.

[21] Caliley Bien. San Marcos will stop adding fluoride. Available at : http://kxan.com/2015/11/11/san-marcos-will-stop-adding-fluoride-to-water-on-nov-12/. Accessed 8 June 2019.

[22] Fluoride action network. Available at: https://fluoridealert.org/issues/water/. Accessed 8 June 2019.

[23] Is Fluoride Bad for You? Or Is Adding Fluoride to Water A Good Thing? Available at https://foodrevolution.org/blog/fluoride-in-water-dangers/. Accessed 8 June 2019.

[24] Kuthy RA, Heller KE, Riniker KJ, McQuistan MR, Qian F (2007). Students’ opinions about treating vulnerable populations immediately after completing community-based clinical experiences. J Dent Educ.

[25] Weeks WB, Wallace AE (2008). Rural-urban differences in primary care physicians’ practice patterns, characteristics, and incomes. J Rural Health.

[26] Lopez-Cepero M, Amini H, Pagano G, Casamassimo P, Rashid R (2013). Advocacy practices among U. S. Pediatric dentists. Pediatr Dent.

